# Supplementary material for: Development and Validation of the Meiji Nutritional Profiling System per Serving Size
Source: Nutrients. 2024 Aug 14;16(16):2700. doi: 10.3390/nu16162700 (PMC11357180; doi:10.3390/nu16162700)
Supplement: Supplementary file 1 [file nutrients-16-02700-s001.zip › nutrients-3145034-supplementary.pdf]

Supplementary materials

S1. Meiji NPS for older adults per serving size

The range for older adults was slightly broader than that for adults (from -53.1 to 129.0). In terms of central tendency, the median Meiji NPS score was 10.2.

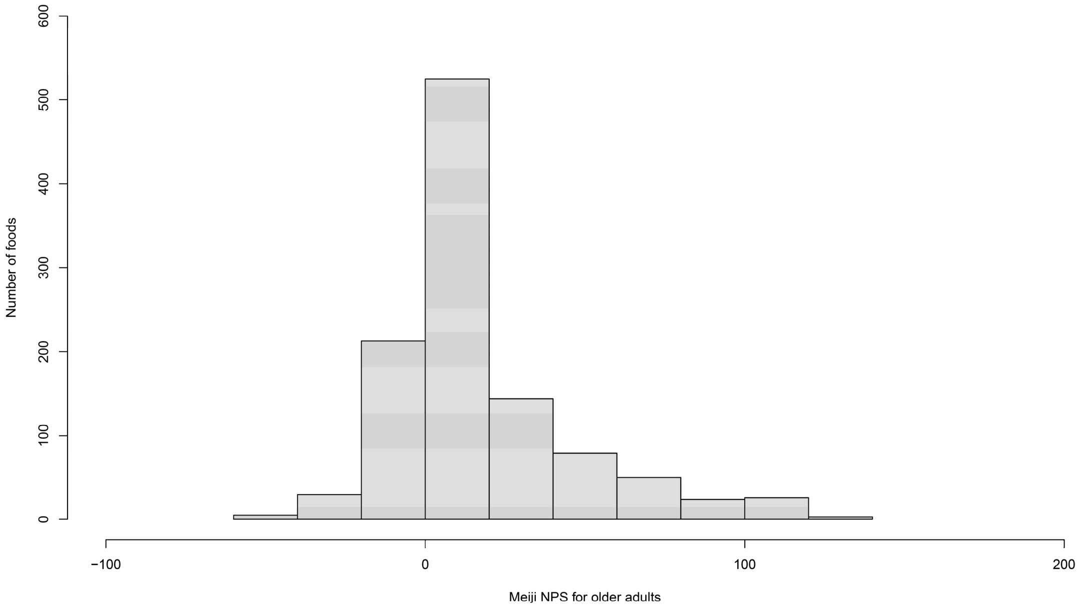

Supplementary Figure S1 Distribution of the Meiji NPS scores for older adults per serving size. The highest score was 129.0, and the lowest score was -53.1.

Supplementary Table S1 Summary results of the Meiji NPS for older adults per serving size.

Items	n	Median	Max	Min	IQR
Cereals	77	0.8	29.4	-53.1	-2.3 to 6.6
Potatoes and starches	18	5.6	22.1	-13.9	3.4 to 7.8
Sugars and sweeteners	0	NA	NA	NA	NA
Pulses	42	47.4	82.0	6.0	32.3 to 62.7
Nuts and seeds	22	24.0	45.1	3.3	11.7 to 28.4
Vegetables	123	13.0	37.3	-0.1	7.4 to 18.0
Fruits	62	10.9	54.1	-15.5	2.3 to 26.8
Mushrooms	24	9.4	61.4	-1.8	7.3 to 13.4
Algae	9	4.7	15.6	-3.4	1.5 to 10.0
Fish and seafood	350	27.2	129.0	-40.3	6.1 to 59.7
Meat	210	8.8	24.1	-10.2	0.5 to 11.8
Eggs	13	7.4	34.3	-1.9	2.3 to 13.4
Milk and milk products	37	18.9	64.2	-6.7	-0.4 to 31.1
Fats and oils	4	-2.1	8.4	-5.2	-3.0 to 0.7
Confectionery	82	-13.2	11.6	-43.0	-22.8 to -5.0
Beverages	10	-8.7	25.2	-13.8	-13.5 to -3.4
Seasonings and spices	16	-7.6	2.4	-29.7	-11.7 to -0.7
Total	1099	10.2	129.0	-53.1	1.1 to 24.7

The Meiji NPS score per serving size for older adults was calculated at 1099. The food categories were based on the food groups listed in the Japanese Food Standard Composition Table 2020 (8th Edition). n, number of food items; Max, maximum; Min, minimum; IQR, interquartile range; NA, not applicable.

Supplementary Table S2 Spearman's correlation coefficients between the Meiji NPS for older adults per serving size and NRF9.3.

Items	n	r	p-values
Cereals	77	0.82	<0.001
Potatoes and starches	18	0.83	<0.001
Sugars and sweeteners	0	NA	NA
Pulses	42	0.40	0.008
Nuts and seeds	22	-0.30	0.160
Vegetables	123	0.02	0.792
Fruits	62	0.25	0.054
Mushrooms	24	0.35	0.090
Algae	9	0.33	0.385
Fish and seafood	350	-0.15	0.006
Meat	210	0.72	<0.001
Eggs	13	0.66	0.017
Milk and milk products	37	0.75	<0.001
Fats and oils	4	0.80	0.333
Confectionery	82	0.47	<0.001
Beverages	10	0.89	0.001
Seasonings and spices	16	0.88	<0.001
Total	1099	0.45	<0.001

Sugar and sweetener data were not available. NPS, nutritional profiling system; NRF, nutrient-rich foods index; NA, not available.

S2. Convergent validity between the Meiji NPS per serving size and HSR

Spearman's correlation coefficient was used to compare the systems. The Spearman's correlation coefficients were 0.51 for adults and 0.50 for older adults, indicating a moderate correlation. However, the correlation coefficients for pulses, nuts and seeds, vegetables, fruits, mushrooms, algae (excluding older adults), and eggs were relatively low and not significant.

Supplementary Table S3 Spearman's correlation coefficients between the Meiji NPS per serving size and HSR.

Items	n	For adults		For older adults	
		r	p-values	r	p-values
Cereals	77	0.73	<0.001	0.79	<0.001
Potatoes and starches	18	0.63	0.005	0.64	0.005
Sugars and sweeteners	0	NA	NA	NA	NA
Pulses	42	0.04	0.792	0.06	0.707
Nuts and seeds	22	-0.10	0.644	-0.24	0.281
Vegetables	123	0.06	0.508	0.10	0.281
Fruits	62	-0.13	0.301	0.00	0.978
Mushrooms	24	0.32	0.123	0.43	0.038
Algae	9	0.41	0.272	0.39	0.305
Fish and seafood	350	0.27	<0.001	0.26	<0.001
Meat	210	0.88	<0.001	0.85	<0.001
Eggs	13	-0.19	0.535	-0.15	0.631
Milk and milk products	37	0.74	<0.001	0.80	<0.001
Fats and oils	4	0.74	0.262	0.26	0.742
Confectionery	82	0.36	<0.001	0.15	0.190
Beverages	10	0.74	0.015	0.88	<0.001
Seasonings and spices	16	0.88	<0.001	0.85	<0.001
Total	1099	0.51	<0.001	0.50	<0.001

NPS, nutritional profiling system; HSR: health star rating; NA, not available.

S3. 0–100 scaled Meiji NPS per serving size

Scores for the Meiji NPS for adults per serving size were scaled between 0 and 100. Specifically, scores below -40 were converted to 0, whereas scores above 40 were converted to 100. The same scaling criteria were applied to the Meiji NPS for older adults. Convergent validity was assessed using Spearman's correlation coefficient between the Meiji NPS and the 0-100 scaled Meiji NPS (Supplementary Table 4). The correlation coefficient was 1.00 for both adults and older adults in total foods.

Supplementary Table S4 Spearman's correlation coefficients between the Meiji NPS per serving size and the 0-100 scaled Meiji NPS per serving size.

Items	n	For adults		For older adults	
		r	p-values	r	p-values
Cereals	77	1.00	<0.001	1.00	<0.001
Potatoes and starches	18	1.00	<0.001	1.00	<0.001
Sugars and sweeteners	0	NA	NA	NA	NA
Pulses	42	0.89	<0.001	0.91	<0.001
Nuts and seeds	22	1.00	<0.001	1.00	<0.001
Vegetables	123	1.00	<0.001	1.00	<0.001
Fruits	62	1.00	<0.001	1.00	<0.001
Mushrooms	24	1.00	<0.001	1.00	<0.001
Algae	9	1.00	<0.001	1.00	<0.001
Fish and seafood	350	0.98	<0.001	0.97	<0.001
Meat	210	1.00	<0.001	1.00	<0.001
Eggs	13	1.00	<0.001	1.00	<0.001
Milk and milk products	37	0.99	<0.001	1.00	<0.001
Fats and oils	4	1.00	0.083	1.00	0.083
Confectionery	82	1.00	<0.001	1.00	<0.001
Beverages	10	1.00	<0.001	1.00	<0.001
Seasonings and spices	16	1.00	<0.001	1.00	<0.001
Total	1099	1.00	<0.001	1.00	<0.001
